# A Healthy Eating Education Program for Midwives to Investigate and Explore Their Knowledge, Understanding, and Confidence to Support Pregnant Women to Eat Healthily: Protocol for a Mixed-Methods Study

**DOI:** 10.2196/resprot.9861

**Published:** 2018-05-25

**Authors:** Shwikar Mahmoud Etman Othman, Mary P Steen, Rasika Jayasekara, Julie-Anne Fleet

**Affiliations:** ^1^ Obstetrics and Gynaecology Nursing Department Faculty of Nursing South Valley University Qena Egypt; ^2^ School of Nursing and Midwifery Division of Health Sciences University of South Australia Adelaide Australia

**Keywords:** healthy eating, midwives, education program, mixed-methods research, pregnancy, study protocol

## Abstract

**Background:**

Nutrition and healthy eating behaviors during pregnancy are vitally important for the health of a mother and her developing baby. However, some midwives have reported a lack of evidence-based nutrition knowledge for providing information about healthy eating to women during pregnancy.

**Objective:**

In this study, the aim is to design and evaluate a healthy eating education program to enhance midwives’ knowledge, understanding, and confidence to support pregnant women in South Australia to make healthy eating choices.

**Methods:**

This mixed-methods study consists of two phases. The first phase, Phase 1, consists of an education program for midwives, “Healthy Eating in Pregnancy,” to be delivered through a workshop or webinar. Each midwife will attend one workshop or webinar, which will be approximately two hours in length. This program will be evaluated through pre-, immediate-, and post-educational questionnaires utilizing a website specifically designed for this study. The participants will be midwives who are members of the Australian College of Midwives and the Australian Nursing and Midwives Federation, and users of social media (eg, Facebook and Twitter) residing and employed in South Australia. Phase 2 will consist of semistructured interviews with a purposive sample of midwives. These interviews will be undertaken to gain an in-depth understanding of midwives’ views and how confident they feel educating pregnant women after receiving the healthy eating education. Interviews will be face-to-face or conducted by telephone with midwives who have participated in the healthy eating educational program.

**Results:**

A systematic review has previously been undertaken to inform this study protocol. This paper describes and discusses the protocol for this mixed-methods study, which will be completed in April 2019.

**Conclusions:**

The results from the systematic review suggest that there is clear justification to undertake this mixed-methods study to investigate and explore midwives’ knowledge, understanding and confidence to support healthy eating in pregnant women. The results and conclusions from the systematic review provided some guidance for the design and development of this study protocol. This mixed-methods study will address a gap in the literature. The results from quantitative and qualitative data sources in this proposed study will help to draw conclusions to address the research topic.

**Registered Report Identifier:**

RR1-10.2196/9861

## Introduction

### Overview of Influences for a Healthy Pregnancy

Cumulative evidence demonstrates a pregnant woman's health behaviors have a life-long influence on her health, and that of her developing baby. Good maternal and fetal outcomes have been associated with healthy nutritional habits and an active lifestyle during pregnancy [1,2]. Poor pregnancy outcomes such as preterm birth and small or large for gestational age are often associated with maternal body mass index disorders and life style choices, for example, being underweight or overweight, as well as smoking and alcohol consumption which are modifiable risk factors [3-6]. Unhealthy maternal behavior in pregnancy has been shown to have long-term effects on children and has been associated with conditions such as cognitive defects, obesity, asthma, and cardiovascular diseases [7-11]. Therefore, nutritional education during pregnancy has an important role in maintaining a healthy status for pregnant women.

### Health Education During Pregnancy

Pregnancy is an opportunity to develop or maintain healthy behaviors, as most pregnant women will be motivated to gain more knowledge in order to give their developing fetus the best start in life [12]. Generally, midwives provide health education for pregnant women during antenatal visits. During antenatal visits, some women prefer verbal advice rather than written information from midwives [13]. It is good practice to first verbally discuss and give an explanation about healthy eating in pregnancy, followed by providing written information to help women understand more clearly. Therefore, written material is a complementary source of information [12]. Midwives have an important public health role and provide health education to pregnant women and new mothers.

### Midwives’ Public Health Role

National and international maternity policies value and support the role of midwives in public health [14]. Health care providers, such as midwives, have a unique opportunity to support women to make choices to promote health and prevent illness [14]. To achieve this goal the emphasis of midwifery policy and practice is to build relationships with the women they care for and to focus on health promotion as a holistic approach [14]. Therefore, the current study will examine the midwives’ role to promote healthy eating, as well as develop and evaluate an educational program for midwives to complement their public health role.

### Significance of Nutritional Health Education Programs

A systematic review is being conducted which aims to examine the effectiveness of healthy eating education programs for improving midwives’ level of knowledge and confidence in promoting healthy eating in pregnant women [15]. This review will consider studies that evaluate diet and nutritional education programs, or training targeted for midwives and student midwives (hospital or community-based midwives, obstetric nurses, birth attendants, doulas, midwives alone or with other health professionals) to provide healthy eating knowledge and advice for pregnant women. The education and training program proposed in this mixed-methods study will be a structured program therefore the considered studies in the review may have a defined period, facilitated as a workshop or seminar. The education and training program can be provided by any health care professionals such as dietitians, nutritionists, midwives, health educators, or any other accredited personnel. The primary outcomes of the systematic review are as follows: (1) level of knowledge acquired by midwives and student midwives regarding diet and nutritional requirements in pregnancy measured by any scale or questionnaire and (2) the level of confidence acquired by midwives and student midwives regarding diet and nutritional requirements in pregnancy measured by any scale or questionnaire.

The systematic review was conducted using a three-stage comprehensive search of seven electronic databases and grey literature. Two independent reviewers assessed each paper prior to inclusion using the standardized critical appraisal instruments for evidence of effectiveness developed by the Joanna Briggs Institute. Preliminary results from this systematic review provided guidance for the design of this mixed-methods study and the development of a semistructured questionnaire to be used at the three-time points during the study. The questionnaire will gather data to investigate midwives’ level of knowledge and confidence to provide healthy eating education and support to pregnant women. The literature highlighted that there is a lack of studies exploring the role of midwives in antenatal nutritional health education [16-18]. The lack of evidence, therefore, justifies exploring the public health role of midwives to address the nutritional health education of pregnant women and their developing baby. Previous studies have shown that some midwives self-reported a lack of basic knowledge of nutrition requirements during pregnancy [16,17]. A cross-sectional study that investigated Australian midwives’ nutrition knowledge, attitudes, and confidence to provide nutrition education during their undergraduate studies and after registration found that some midwives did not receive evidence-based nutritional health education [16]. It is, therefore, important to investigate the needs of midwives to improve the way in which nutritional health education is provided to pregnant women.

### Aim and Objectives

This paper describes the study protocol for a healthy eating education program during pregnancy. The aim is to design and evaluate a healthy eating education program to enhance midwives’ knowledge, understanding, and confidence to support South Australian pregnant women to eat healthily during and after their pregnancy. The study will address the following research objectives through two phases.

Phase 1 will assess midwives’ knowledge and level of confidence when providing information on diet and nutrition education for pregnant women and evaluate a healthy eating education program.

Phase 2 will explore midwives’ views on how they provide advice on healthy eating for pregnant women, through an individual interview after they have attended a healthy eating education program.

## Methods

### Study Design

This proposed research study protocol will utilize a sequential explanatory mixed-methods approach guided by a conceptual framework and undertaken in two phases, namely a quantitative phase (Phase 1), followed by a qualitative phase (Phase 2).

### Conceptual Framework

**Figure 1:**
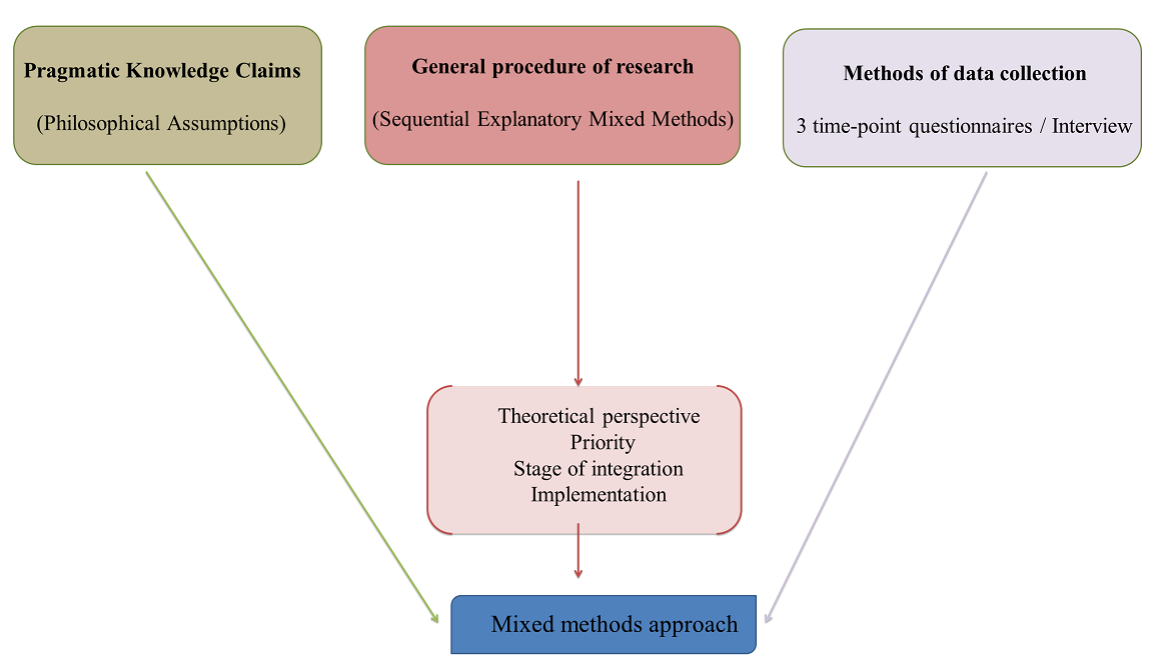
Three framework elements of inquiry for a mixed-methods approach developed from Creswell [22].

The framework elements in this mixed-methods study are philosophical assumptions (pragmatic knowledge claims), strategies including Sequential Explanatory Mixed-Methods Design, and methods (to describe data collection procedures). These are summarized in Figure 1.

#### Philosophical Assumptions: Pragmatic Knowledge Claims

Pragmatism is considered as a worldview, which accepts multiple realities and supports practicality when addressing the research question and it reflects both biased and unbiased perspectives. This pragmatic perspective as a philosophical methodology draws on utilizing “what works,” utilizing different aspects of this, setting priority to the value of the research problems and questions, and gathering both objective and subjective data [19].

#### Strategies Including Sequential Explanatory Mixed-Methods Design

**Figure 2:**
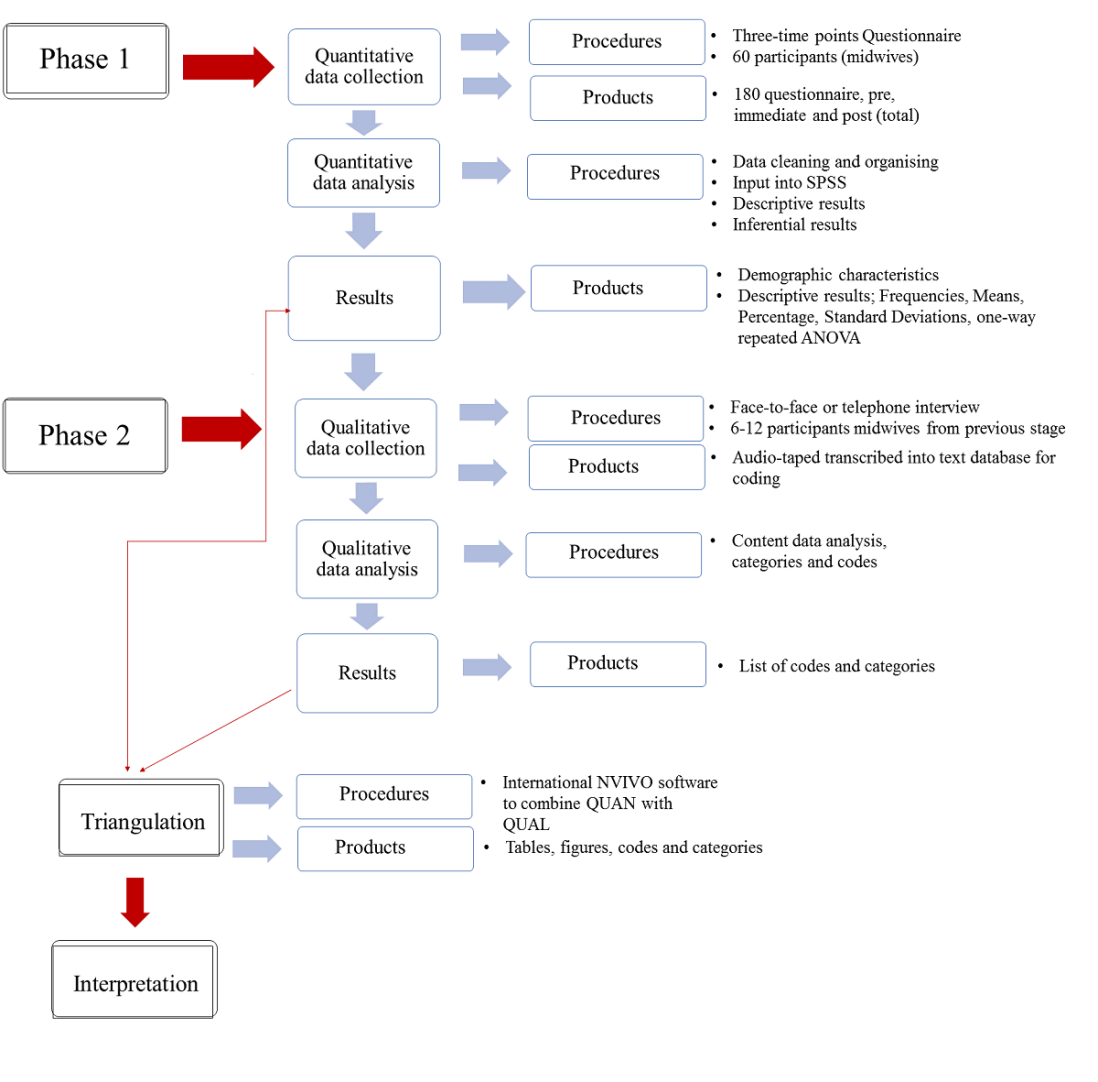
Sequential Explanatory Mixed-Methods Design adapted from Jirojwong, Johnson and Welch [21] and Creswell [20]. ANOVA: analysis of variance; QUAL: qualitative data; QUAN: quantitative data; SPSS: Statistical Package for Social Sciences.

This proposed research study will use a Sequential Explanatory Mixed-Methods Design according to Creswell [20]*.* The two phases will gather both quantitative and qualitative data in a sequential manner. The first phase involves collecting and analyzing quantitative descriptive (numeric) data, which gives a general picture and overview of the data related to the research problem. The second phase involves collecting and analyzing qualitative (text) data, which will help explain the general overview and explore the midwives’ views in more depth. The interpretation of results is usually undertaken following completion of the second phase. Figure 2, adapted from Jirojwong, Johnson and Welch [21] and Creswell [20], describes this process. Finally, the data obtained from the two phases are integrated [22] to draw a comprehensive and accurate conclusion. The research outcomes using this approach provide a broader, more comprehensive picture of a specific phenomenon compared to using either a quantitative or qualitative method alone [23-25].

#### Methods of Data Collection

A semistructured questionnaire will be used in Phase 1 to collect data. The questionnaire was designed and adapted from the literature and previous applied questionnaires [16,18,26] to examine healthy eating and nutrition during pregnancy, as well as problems related to nutrition during pregnancy [17,27-41]. This questionnaire will be used to collect data at 3 time points.

The semistructured, pre-educational questionnaire used in this study included 5 sections asking midwives for their previous nutrition education, level of confidence, nutrition knowledge, an invitation to attend the workshop or webinar, and demographic characteristics based on a previously validated questionnaire (used after seeking permission from the author) [16]. For the immediate- and post-educational questionnaires, midwives will only be asked about their level of confidence and nutrition knowledge.

A pilot of the questionnaire will be undertaken to ascertain validity and reliability of the instrument. Questionnaire validity refers to the extent to which it measures what the questionnaire is intended to measure. Measuring content validity assesses whether the instrument adequately covers all the content with respect to the variables and it is measured by asking experts (face validity testing) their opinion about whether an instrument measures the concept intended [21,42]. The expert panel will be consulted and invited to comment on the questionnaire to give feedback on the instrument (ie, on wording and the order of the questions). Three midwifery experts and 2 dietitians will be invited to review the questionnaire and feedback will be obtained on the clarity of the instrument (questionnaire).

A common method used to estimate the reliability of a measurement is the “test-retest” method, which involves the same test being given to the same participants more than once under the same conditions to evaluate if the responses to the test reflect true variables [42,43]. This questionnaire will undergo a pilot using the “test-retest” method with the aim of assessing whether the same results will be obtained, and therefore its reliability. The pilot will involve three to five midwifery researchers and students who will be invited to participate in the pilot and will not be involved in the main study. The midwifery researchers and students involved in the pilot will complete the pre-educational questionnaire twice, once immediately and then again one week later.

Phase 2 of this study will be initiated after the completion of Phase 1. It will consist of a semistructured face-to-face or telephone interview to be scheduled 8 weeks after attendance of the Healthy Eating Education Program. The designed interview schedule will be piloted with three to five midwives or student midwives, and any suggestions or recommendations obtained during the pilot will be incorporated into the final version of the interview schedule.

### Phase 1 Methodology: Designing, Facilitating, and Evaluating the Healthy Eating Education Program

#### Designing the Workshop or Webinar

The workshop or webinar will be designed based on results gathered from the systematic review and utilize material from the “EatWell” Assist program [44] and will be designed based on the following criteria: (1) the healthy eating educational program will incorporate evidence-based guidelines and (2) explain the significant nutritional elements including sources, importance, and recommended intake or amount. Furthermore, a workbook will be designed for workshop activities and recommended reading.

##### Study Website

The study website will include detailed information about the study. The home page will introduce the study and clearly state its aim and objectives. Other webpages will provide information on how to participate in the study, the ethical considerations related to the study, the research team, and how to contact the primary researcher. Relevant webpages will give prospective participants the option of attending a workshop or webinar, provide online questionnaires to complete, and provide an invitation to attend a follow up face-to-face or telephone interview.

##### Recruitment

Midwives residing and employed in South Australia will be invited to participate in the study. An invitation will be sent via the Australian College of Midwives (ACM) and the Australian Nursing and Midwifery Federation (ANMF, South Australian Offices) e-bulletins. A dedicated study website will be designed and utilized with a domain name (http://healthyeating educationformidwives.com/) for midwives to access and participate in the study. An internet advertisement using Google search engine and social media outlets such as Facebook and Twitter will be linked to the study website. A study Facebook page and Twitter account will be set up to increase awareness of the study.

#### Pre-educational Workshop or webinar Questionnaire to Investigate Midwives’ Knowledge and Level of Confidence Regarding Healthy Eating

A pre-educational healthy eating questionnaire will be undertaken to investigate the midwives pre-existing knowledge, understanding, and level of confidence prior to attending the healthy eating education program.

##### Settings

The setting for the participant workshop will be the University of South Australia, School of Nursing and Midwifery, City Campus, Adelaide, or other University of South Australia Campuses, such as Whyalla or Mount Gambier, if this is preferred. For participants who have a preference to attend online or are unable to attend a workshop, an online webinar will be offered.

#### Implementation of Healthy Eating Education Program

The healthy eating education program will be delivered by attendance at a workshop or webinar (online virtual classroom).

The first author, SO, will have cofacilitation support to introduce and facilitate a “Healthy Eating in Pregnancy” education program (workshop or webinar) for midwives. The workshop duration will be approximately two hours (including time for refreshment and completing the posttest educational questionnaire). The guide used for the contents covered in the workshop and webinar is described in Textbox 1.

**A guide for content to be covered in the Healthy Eating Education Program workshop or webinar.**

- Healthy eating and dietary requirements
- Macronutrients (protein, carbohydrate, fibers, Omega 3 and fats and essential fatty acids), micronutrients (include vitamins A, C, E and B complex and minerals [Se, Fe, Ca, Mg, and Zn]), folic acid, fish consumption and iodine with pregnancy. The aim of this information is to improve the participant’s knowledge about role and sources and how it can enhance health and reduce risk.
- Portion sizes
- Eating myths
- Vegetarians and vegans eating
- Cultural food choices
- Eating habits and behaviors
- Preparing food and hygiene safety
- Physical activity
- Dental care during pregnancy
- Probiotics and prebiotics

The teaching materials will include a workbook, quiz, brochure, and recommended reading.

#### Posteducational Workshop and Webinar Questionnaire

The same pre-educational questionnaire administered to the midwives prior to attendance of the workshop or webinar will be completed again at the end of the workshop or webinar to evaluate the midwives' healthy eating knowledge immediately after the education program. This questionnaire will be provided in a PDF format (for the workshop) and online via the study website for the webinar (virtual classroom), according to the midwives’ preference. The researcher and workshop facilitator will remind the midwives that they will be contacted in six to eight weeks after attending the educational program to complete a final questionnaire (using the same pre-educational questionnaire).

#### Follow-up Educational Workshop or Webinar Questionnaire

Six to eight weeks after attending the workshop or webinar, the primary researcher will contact the midwives by telephone or email and ask them to complete the final posteducational questionnaire.

#### Participants or Population

Midwives residing and employed in South Australia who are members of the ACM and ANMF will be invited to participate in the study. Australian social media groups such as the ACM and ANMF groups on Facebook and Twitter will be provided with a link to the study website.

#### Sample Size and Power Calculation

A single-factor, repeated measures design with a sample of 5 subjects, measured at 3 time points, achieves a 91% power to detect differences among the means using a Geisser-Greenhouse Corrected *F* Test at a .05 significance level. The SD across subjects at the same time point is assumed to be 25. The pattern of the covariance matrix is to have all correlations equal with a correlation of .70 between the first and second time point measurements. The SD of the hypothesized means is 17.00. The expected mean scores over the 3 time points are 50, 90 and 80.

Based on a power calculation, only a very small number of midwives (n=5) would be required to demonstrate the expected large increase in knowledge and level of confidence as a result of the healthy eating education program (workshop or webinar). However, we will recruit a larger number of participants (n=60) to ensure that we have midwives covering abroad range of age, experiences, and locations.

#### Data Analysis

Data from the pre-educational questionnaire will be entered into the Statistical Package for Social Sciences (SPSS) IBM version 25. Descriptive analysis will be used to examine the midwives’ demographic characteristics, such as age, level of education, years of experience, type of maternity services, previous education related to nutrition, and place of practice. The results will be used to describe and summarize the data collected. Data will be presented as frequencies, means, SDs, and percentages. Data related to the level of knowledge and confidence will be analyzed using mixed-effect models to examine the variance in nutritional knowledge and level of confidence over the 3 time points.

Descriptive analysis of questionnaire data will be carried out using an ordinal (1-5 point) Likert scale with the range from ‘very confident’ (5) to ‘not confident at all’ (1). Spearman’s rank correlations coefficient will be used to evaluate the strength and relationship between two or more variables. Inferential statistics used for making conclusions will compare differences and associations using analysis of variance (ANOVA) tests [25]. Open-ended question responses will be coded as positive or negative, and for content analysis to compare different categories at different time periods [45]. A bio-statistician will be available to assist with the analysis. All questionnaire responses will be numerically coded using a predefined coding scheme.

### Phase 2 Methodology: Exploration of Midwives’ Views of the Healthy Eating Education Program

Eight weeks after the Healthy Eating Education Program, a small sample of midwives who participated in the program (12 or less) will be invited to participate in a semi-structured interview either conducted face-to-face or by telephone. An interview schedule will guide the interviews, which will take approximately 30-60 minutes and obtain the participants’ views on the healthy eating education they received. The semi-structured interview will help the midwives to express their feelings freely and enable the researcher to encourage midwives to expand more on what is being discussed. However, the researchers will need to remain objective and open to the possibility that the data may be different than expected [25]. The interview guide will be developed from the results of the systematic review (included studies) and findings of Phase 1.

#### Selection of Participants for Phase 2

A purposive sample of midwives (approximately 12 or less) will be interviewed 8 weeks after completing the Healthy Eating program. Data from participants’ interviews will be recorded and transcribed verbatim. The transcripts will be analyzed on an individual basis until data saturation is reached and recruitment will then cease. The selection of midwives will be based on midwives who work in urban and rural (South Australia) antenatal clinics, Midwifery Group Practices and antenatal parent education classes.

#### Data Analysis in Qualitative Research

Content analysis will be used to evaluate the semistructured interview responses. This method is used for identifying, analyzing, and reporting concepts or categories within data. This analysis method minimally organizes and describes data sets in detail. Content analysis as a research strategy is a methodical and systematic method for describing and evaluating phenomena, which allows for testing theoretical issues to establish understanding of data and comprehension of information [46,47]. The aim of utilizing content analysis will be to achieve a broad description of midwives’ views after receiving the healthy eating education.

#### Content Analysis Process

Interview content analysis will be based on the Conventional content analysis approach which will assist to gain more insights on healthy eating in pregnancy education for midwives after they have received the training [48]. The 3-phase framework for content analysis as described by Elo and Kyngas [45] will be utilized as described below.

##### Preparation Phase

This phase commences with selecting the unit of analysis, this unit can be a word or a theme, relevant to the research question. Using the manifest content where the researcher describes what information was actually said (ie, uses the same words and describes the visible and what is said in the text) [49].

##### Organizing Phase

After making sense of the data, an inductive approach will be used starting from open coding for whole reading of the dataset, creating grouping and categories, then abstraction to formulate a general description of the research topic through generating main categories, generic categories, and subcategories from analyzed data.

##### Reporting the Analyzing Process and the Results

A final report will be written to provide brief, classified, logical, varied, realistic, and interesting conclusions from the story obtained from the data obtained, within, and across, all codes and categories.

## Results

### Triangulation of Quantitative and Qualitative Data Findings

The sequential explanatory design enables the reporting of participants’ cases as one continued story starting from the pre-educational questionnaire, then the healthy eating education workshop or webinar, continued to the participants attending an interview based on the findings from the systematic review and finally to make general conclusions about the participants’ knowledge, level of confidence views, and understanding their role in healthy eating education. According to the conceptual framework (Figure 1) and the sequential explanatory design discussed in Figure 2, an initial data analysis will be undertaken when completing the systematic review. Moreover, this analysis will continue during Phase 1 (quantitative data extracted from questionnaires with closed- and open-ended questions). The results of the systematic review and the quantitative phase will help to develop a guide for the semi-structured, in-depth interviews with midwives. Semi-structured interview data will be analyzed using content analysis, and the results outcome from this phase will be integrated together with the previous analysis to obtain a broader and more accurate picture describing midwives’ nutritional knowledge and confidence.

### Ethical Considerations

Ethics approval has been granted from the Human Research Ethics Committee at the University of South Australia. Approval has been given to advertise the study via the ACM and the ANMF professional bodies to invite midwives to participate in the study. The program will also be recognized by the ACM for continual professional development points. The link to the study website will be included. The study website will also be linked to the Australian Midwives social media groups such as the ACM and ANMF groups on Facebook and Twitter. To maintain confidentiality, a unique identifying number (participant ID) will be assigned to the data collected for each participant. Participation in the study is voluntary and contact details of the research team and a person who is responsible for receiving any complaints will be provided on the study website.

Participants will be able to access a participant information sheet that will outline the purpose of the study, the phases and its importance, via the study website. Participation is voluntary, and midwives can withdraw at any time without influencing their status now or in the future. The participants will be informed about the upcoming workshops and interviews if they wish to participate, and interviews will be audiotaped for further analysis. The transcripts and tape will be securely stored in a locked cabinet in a locked room and only the research team will have access to it.

The results will be nonidentifiable, and findings will be published in journals and at conferences. Participants will be able to access a summary of the findings from the study website. Anticipated benefits of the study include an increase in the level of knowledge for midwives regarding nutritional healthy eating education. Midwives who participate in the health education program (workshop or webinar) will be given two points of Continuing Professional Development on completing the education program through the ACM.

## Discussion

### Study Rationale

It is anticipated that there will be several expected benefits from undertaking and participating in this proposed research. For example, midwives who reside in South Australia will have an opportunity to attend a healthy eating education program (either as a workshop facilitated on campus at the University of South Australia and also accessible as an internet-based webinar) which will be freely available to access and be validated through this research study. This healthy eating educational opportunity has the potential to enhance the knowledge and confidence of midwives who reside in South Australia. It is envisaged that participation in this study will improve midwives' awareness and knowledge of evidence-based guidelines regarding healthy eating in pregnancy. In addition, midwives’ confidence may increase to enable them to provide additional nutritional healthy eating education and support for pregnant women. Should this program increase the midwives’ knowledge and confidence to educate women, this may improve maternal and fetal outcomes.

### Conclusion

The preliminary results of the on-going systematic review suggest that there is clear justification to undertake this mixed-methods study to investigate and explore midwives’ knowledge, understanding and confidence to support pregnant women to eat healthily. Findings and conclusions from this systematic review has provided some guidance for the design and development of this protocol. The questionnaire has been designed to be used at 3 time points during the first phase of this research study. A follow up second phase will provide an opportunity to gain a more in-depth understanding of midwives’ views after they have received the healthy eating education program. The results from both phases (quantitative and qualitative) data sources will help to draw conclusions to address the research topic.

## Appendix

Multimedia Appendix 1 Peer-reviewer report.

## Abbreviations

ACM: Australian College of Midwives
ANMF: Australian Nursing and Midwifery Federation
ANOVA: analysis of variance
SPSS: Statistical Package for Social Sciences
