# Supplementary material for: A Healthy Eating Education Program for Midwives to Investigate and Explore Their Knowledge, Understanding, and Confidence to Support Pregnant Women to Eat Healthily: Protocol for a Mixed-Methods Study
Source: JMIR Res Protoc. 2018 May 25;7(5):e143. doi: 10.2196/resprot.9861 (PMC5993976; doi:10.2196/resprot.9861)
Supplement: Multimedia Appendix 1 [file resprot_v7i5e143_app1.pdf]

University of South Australia | School of Nursing and Midwifery

Confirmation of Candidature

Panel Assessment Form

**Name of Candidate**	**Shwikar Mahmoud Etman Othman**
**Name of Panel Member**	**Tahereh Ziaian**

My opinion of the students' proposal is summarised below *[Tick where appropriate]*

**Topic**	**Satisfactory**	**Unsatisfactory**
The topic contributes new knowledge to the subject area	X	
The topic has sufficient scope for doctoral research	X	
The proposed research has a sound policy, philosophical, scientific or theoretical base	X	
The proposed application of the research to issues in health and health care is discussed	X	
To my knowledge, the material in this proposed research has not been published before	X	

**Literature Review**	**Satisfactory**	**Unsatisfactory**
Succinct summary of the relevant literature was provided in the proposal	X	
The literature cited by the student is based chiefly on primary sources	X	
The sources of evidence on which the proposed research is based are clearly stated	X	
The interpretations and conclusions are justified by the evidence presented	X	

**Proposal**	**Satisfactory**	**Unsatisfactory**
The title of the research accurately reflects the content of the proposal	X	
Clear and logical description of proposed research is presented	X	
The writing style is grammatically correct and references are cited appropriately and correspond accurately to the conventions used by the School	X	
A 300 word abstract is included in the proposal that accurately reflects the proposed research	X	
The research proposal does not exceed 20 pages		X
Sufficient detail of proposed research is provided to allow evaluation	X	

**Research Method**	**Satisfactory**	**Unsatisfactory**
There is a clear statement of the purpose, aim, question or hypothesis of the research	X	
All key concepts are clearly defined (as appropriate)	X	
Research participants are clearly described.<br>*The description covers (as appropriate) the inclusion criteria, identification and recruitment of participants, and justification of sample size*	X	
Data collection process is clearly described.<br>*The description covers (as appropriate) what data will be collected, how the data will be collected and the processes that will be used to ensure accuracy of data. Proposal includes any data collection tools, scales or instruments that will be used*	X	
The proposed data analysis is clearly described, justified and appropriate	X	
The ethical aspects of the research are addressed (as appropriate)	X	
The ethical approval and any other permissions that are required to conduct research are described	X	
Any resource implications of the proposed research are adequately addressed	X	
Timeframe of the proposed research is described and is appropriate	X	
Trial Table of Contents is appropriate	X	

Are there any cost implications for this proposed study? Yes ☒☐ No ☐

If yes, have these costs been addressed in the proposal? Yes ☒☐ No ☐

Reviewer feedback to candidate

This is a timely project that addresses a gap in the literature. It is a worthy and valuable study which uses a mixed method approach. The proposal is a well thought out program of research and it is likely to achieve the aims it has set out. Well done. However, there are a few minor grammatical errors and some minor issues and clarifications that need to be addressed.

Overall my impression is that this is a well structured and a well conceived research proposal. I have no major concerns with the proposal and my comments throughout the proposal are requests for clarity and in some sections suggestions are made for minor changes, they are not significant flaws. Please see marked up copy with my comments.

I wish the researcher very well with the project.

Kind Regards,

Tahereh

A.Professor Tahereh Ziaian, PhD, MAPS

Panel Members’ Recommendation

☒ **Research proposal requires (minor) revision**
The research proposal is of an appropriate standard but some revisions are required.

☐ **Enrolment is Confirmed**
The research proposal is of an appropriate standard and so enrolment should be confirmed.

☐ **Provisional Enrolment is to Continue**
The research proposal requires major amendments and so provisional enrolment should be continued.

☐ **Candidature to be Suspended**
The proposal was not of an adequate standard and so candidature should be suspended.

Please include additional typed information, comments and critique of the proposal that can be sent to the candidate and supervisors

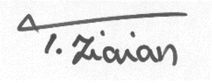

Reviewer signature: ______________________________ Date: 07/09/2017
